# Protocol for evaluating the economic viability of adiabatic compressed air energy storage systems

**DOI:** 10.1016/j.xpro.2026.104382

**Published:** 2026-02-19

**Authors:** Danlei Yang, Yang Wang, Jihong Wang, Zhenhua Rui, Wei He

**Affiliations:** 1 Department of Engineering, King’s College London, WC2R 2LS London, UK; 2 Energy System Catapult, B4 6BS Birmingham, UK; 3 School of Engineering, University of Warwick, CV4 7AL Coventry, UK; 4 College of Carbon Neutrality Future Technology, the China University of Petroleum (Beijing), Beijing 102249, China

**Keywords:** Physics, Energy, Environmental sciences

## Abstract

Techno-economic evaluation is vital for assessing long-duration energy storage. Here, we present a computational workflow evaluating adiabatic compressed air energy storage economic performance. We describe steps for deriving learning-driven cost projections via experience-curve modeling and quantifying uncertainties using Monte Carlo simulations. We then detail procedures for discounted cash flow analysis to determine costs. This protocol enables systematic assessment of storage economic viability.

For complete details on the use and execution of this protocol, please refer to Yang et al.^1^

## Highlights

• Harmonize global CAES cost data across currencies and inflation years
• Derive learning-driven cost projections using Wright’s law modeling
• Quantify experience rate uncertainty via Monte Carlo simulations
• Assess financial viability through a lifecycle DCF and PVR-TCO model

## Abstract (editor highlights)

Publisher’s note: Undertaking any experimental protocol requires adherence to local institutional guidelines for laboratory safety and ethics.

## Graphical abstract

**Figure:**
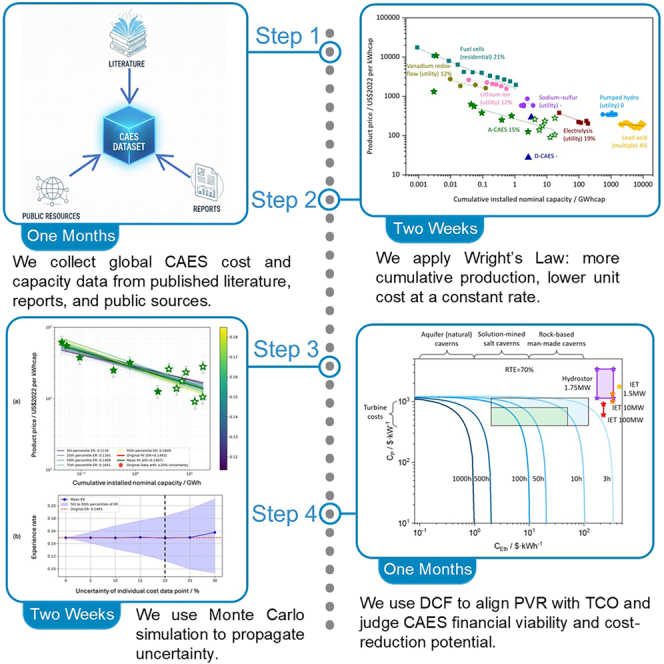

## Before you begin

This protocol is designed for a specific use case: evaluating cost declines and financial feasibility of adiabatic compressed air energy storage (A-CAES) using a global project cost database, Wright’s Law experience-curve fitting, Monte Carlo simulation for data uncertainty, and a lifecycle discounted cash flow (DCF) model. In this protocol, we focus on grid-scale A-CAES projects (≥10 MW) to estimate an experience rate and project future costs, then test economic viability across discharge-duration scenarios. Optional applications include extending the same workflow to other long-duration energy storage technologies (e.g., pumped hydro or flow batteries) if comparable cumulative-capacity and cost datasets are available.

### Step-by-step implementation workflow

1. Define data scope and inclusion rules.
  a. Specify the target technology and market scale as grid-level A-CAES.
  b. Set inclusion thresholds to reduce scale bias; in our study we exclude sub-MW and early pilot projects and retain only ≥10 MW cases.

***Note:*** Keeping a consistent scale band avoids artificially high learning rates driven by small experimental outliers.

2. Harmonize cost units and currencies.
  a. Adjust all reported project costs to a common year (2022) using OECD consumer price indices.
  b. Convert inflation-adjusted local costs to 2022 USD using OECD purchasing power parity indices.
  c. Convert power-based costs/capacities (US$/kW, GW) to energy-based ones (US$/kWh, GWh) using technology-specific C-rates (power-to-energy ratios).

**CRITICAL:** Record every assumption (e.g., C-rate used for conversion) alongside its source so the dataset and learning-curve fit are reproducible.

3. Build the experience-curve dataset.
  a. Order projects chronologically and compute cumulative installed capacity X.
  b. Pair each cumulative capacity with the corresponding unit cost P(X).

**Pause point:** After this step, re-check for obvious data entry errors or extreme outliers before fitting.

Prerequisites: To implement the following workflow, users require a computational environment supporting statistical simulation (e.g., Python, or MATLAB). Users are responsible for collecting and preparing their own baseline cost datasets. Additionally, users are expected to have a foundational background in probabilistic analysis and Monte Carlo methods to interpret the sensitivity and distribution of the outputs.

4. Set uncertainty assumptions for Monte Carlo runs.
  a. Assign an observational/measurement uncertainty range to each cost point; we use ±20% as a baseline.
  b. Choose a noise distribution and sampling method (e.g., independent perturbations) and fix a random seed for repeatability.

***Note:*** You can test sensitivity by widening the assumed uncertainty band (e.g., up to 30%). It should be noted that these specific percentages are not static; they are highly dependent on the scope and nature of the specific work being analyzed. These ranges align with common preliminary estimation standards, where higher volatility is expected in the early stages of a project. Users may adjust these parameters to reflect their specific operational contexts.

5. Prepare DCF model inputs.
  a. Define lifecycle parameters (project term, discount rate, replacement intervals, efficiency, fixed/variable O&M).
  b. Specify revenue streams (e.g., energy arbitrage and capacity payments) and cost components to compute present value of revenue (PVR) and total cost of ownership (TCO).
  c. Set discharge-duration scenarios (e.g., 3–1000 h) with consistent power/energy cost inputs for alignment with the learning-curve outputs.

***Optional:*** When adapting this protocol to other long-duration energy storage technologies, the core PVR–TCO analytical framework and the probabilistic simulation logic are fully transferable, whereas the specific technical performance parameters (e.g., round-trip efficiency, degradation rates) and cost-benefit variables must be re-calibrated to match the target technology.

Once these preparations are complete, proceed to Wright’s Law fitting, Monte Carlo uncertainty propagation, and DCF evaluation.

### Innovation

Our innovation is a reproducible, end-to-end workflow that links experience-curve learning, Monte Carlo uncertainty propagation, and DCF into a single CAES cost-and-viability assessment. Unlike studies that extrapolate costs with a standalone learning curve, we (i) compile a harmonized global CAES cost dataset, standardizing currencies and years, converting power-based to energy-based metrics with explicit C-rate assumptions, and applying scale-based screening to limit size-driven bias; (ii) treat each Wright’s Law cost point as uncertain, injecting observational/measurement error and using Monte Carlo sampling to generate probability distributions for experience rate and future costs rather than a single deterministic fit; and (iii) embed a lifecycle DCF model, aligning PVR with TCO to test financial feasibility and cost-reduction potential across discharge-duration and market scenarios. This creates a closed loop from “technology learning → cost evolution → economic viability.” The protocol is transferable to other long-duration storage technologies, enabling consistent cross-technology comparisons.

### Data collection and harmonization

**Timing: 1 month**

This section details the procedures for gathering and standardizing global CAES cost and capacity data from diverse sources, including published literature, industry reports, and public databases. The objective is to establish a consistent and comparable baseline dataset by harmonizing different currency units and inflation adjustments.

6. Define the study scope and inclusion rules.
  a. Set the target as grid-scale A-CAES (adiabatic CAES) projects.
  b. Define a minimum scale threshold (e.g., ≥10 MW).
  c. Standardize the cost boundary (e.g., CapEx/EPC cost or total installed cost) and output units (2022 USD $/kWh and cumulative GWh).
7. Collect raw project data.
  a. Extract commissioning year, location, power (MW), energy (MWh/GWh), reported cost, currency, and source from academic papers, industry reports, and storage databases.
  b. Enter each project as one row in a master spreadsheet and preserve original values/units and citations for traceability.

**CRITICAL:** Do not alter any raw values or units before conversion so every transformation can be audited.

8. Harmonize all costs to a common year and currency.
  a. Inflate/deflate reported costs to a single base year (e.g., 2022) using national CPI series.
  b. Convert inflation-adjusted costs to 2022 USD using purchasing power parity (PPP) indices.
9. Convert power-based metrics to energy-based metrics.
  a. If a project reports only $/kW, convert to $/kWh using the project’s discharge duration or an explicit C-rate assumption.
  b. If discharge duration is missing, apply a justified default and document the value and rationale.
  c. Convert installed power (MW/GW) to installed energy (MWh/GWh) using the same C-rate.

***Note:*** Use a single conversion logic across projects to avoid biasing the experience curve.

10. Quality-check and freeze the dataset.
  a. Check unit consistency, duplicates, missing years, order-of-magnitude deviations and negative costs.
  b. Separate clear pilot-scale cases into a “pilot subset” if needed.

**Pause point:** After QC, save a read-only frozen version of the dataset for analysis.

### Model setup before analysis

**Timing: 1–2 months**

This section describes the preliminary configuration of computational variables and simulation parameters required for the subsequent analysis. It establishes the mathematical basis for experience-curve fitting, defines the stochastic boundaries for uncertainty propagation, and calibrates the economic inputs for the lifecycle discounted cash flow model. These preparations ensure that all following steps are executed on a consistent and reproducible data foundation.

11. Prepare learning-curve variables for Wright’s Law fitting.
  a. Sort projects by year and compute cumulative installed energy X(GWh).
  b. Pair each cumulative X with its unit cost P(X) (US$/kWh).
  c. Log-transform X and P(X)to create regression-ready inputs.

***Note:*** Keep both a full sample and a ≥10 MW subset if you want to control for scale effects.

12. Specify uncertainty and sampling for Monte Carlo simulation.
  a. Assign an observational/measurement error band to each cost point.
  b. Choose a perturbation distribution and a trial count.
  c. Fix and record a random seed to ensure reproducibility.

**CRITICAL:** Apply the same uncertainty rule to all points to avoid systematic drift in the ER estimate.

13. Set up lifecycle inputs for DCF analysis.
  a. Define project life, discount rate, RTE (round-trip efficiency), replacement intervals, and fixed/variable O&M costs.
  b. Define revenue streams to compute PVR and TCO.
  c. Create discharge-duration scenarios (e.g., 3–1000 h) and keep all scenario costs consistent with learning-curve units and base year.

***Optional:*** If you apply this protocol to another market, swap in that market’s revenue structure while keeping the PVR–TCO framework.

After Preparations 1 and 2, proceed to the main protocol steps: Wright’s Law regression, Monte Carlo propagation, and integrated DCF evaluation.

## Key resources table

**Table:** 

REAGENT or RESOURCE	SOURCE	IDENTIFIER
**Deposited data**		

Historical CAES Cost Data	Academic literature, industry reports, official websites	Available upon request
OECD Consumer Price Indices	OECD database	OECD database: https://stats.oecd.org
OECD Purchasing Power Parity Indices	OECD database	OECD database: https://stats.oecd.org

**Software and algorithms**		

MATLAB R2018b	MathWorks	MathWorks: https://www.mathworks.com
Discounted Cash Flow Analysis Codes	This paper	Zenodo: https://doi.org/10.5281/zenodo.17194806
Monte Carlo Simulation Scripts	This paper	Available upon request

## Step-by-step method details

### Fit a CAES experience curve with Wright’s law and estimate the experience rate

**Timing: 2 weeks**

This part of the protocol describes the application of Wright’s Law to model the relationship between cumulative installed capacity and unit cost reductions. By fitting an experience curve, the framework quantifies the technology’s learning rate, providing a mathematical basis for projecting future cost declines.

This major step fits Wright’s Law to cumulative installed capacity and unit cost, producing an experience curve and baseline experience rate (ER) for cost projection.

1. Prepare regression inputs and verify data integrity.
  a. Load the frozen dataset.
  b. Confirm each project has cumulative capacity *X* (GWh) and unit cost *P*(*X*) (2022 USD/kWh).
  c. Compute ln(*X*) and ln(*P*) and confirm there are no zeros or missing values.
    i. If gaps or outliers appear, return to Preparation one to correct and re-freeze the dataset.
    ii. Flag pilot-scale projects as a separate subset if needed.
2. Fit Wright’s Law^2^ and extract parameters.
  a. Perform an ordinary least squares (OLS) regression on the log-transformed variables: ln(*P*) = ln(*P*_0_)−*b*ln(*X*).
  b. Execute the regression using the MATLAB R2018b.
  c. Record the slope *b* and goodness of fit (e.g., *R*^2^).
  d. Compute the experience rate as *ER* = 1−2^−*b*^.

***Note:*** The log-transformation is used to linearize the power law and stabilize variance (addressing potential heteroscedasticity).

**Figure 1:**
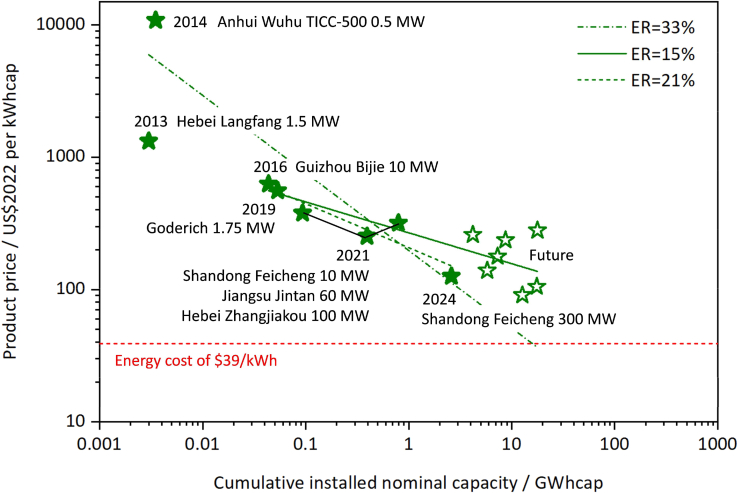
Cost data of A-CAES projects and associated experience rate analysis

**Figure 2:**
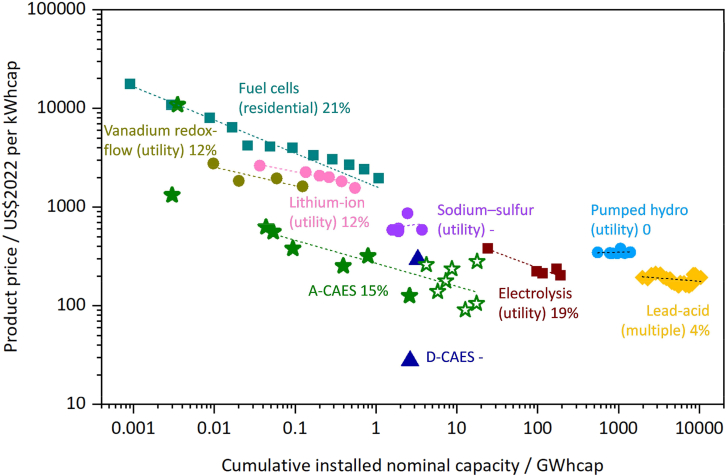
Comparison of CAES and various energy storage technologies based on experience rate (ER) The dashed lines represent the learning curves for various technologies, where the slope of each curve corresponds to the ER.
  c. Export the baseline future cost trajectory.

***Note:*** See troubleshooting 1.

3. Generate the baseline experience curve and cost path.
  a. Transform the fit back to the original scale.
  b. Plot the experience curve (Figures 1 and 2).

### Propagate uncertainty with Monte Carlo simulation to obtain ER and cost distributions

**Timing: 2 weeks**

This section outlines the stochastic modeling approach used to propagate data uncertainties through the cost-reduction projections. By performing Monte Carlo simulations, the protocol identifies the sensitivity of the experience rate to individual data fluctuations and generates a probabilistic range for future outcomes.

This major step applies Monte Carlo simulation to model observational/measurement errors in each cost point, yielding probability distributions for ER and future costs.

**Figure 3:**
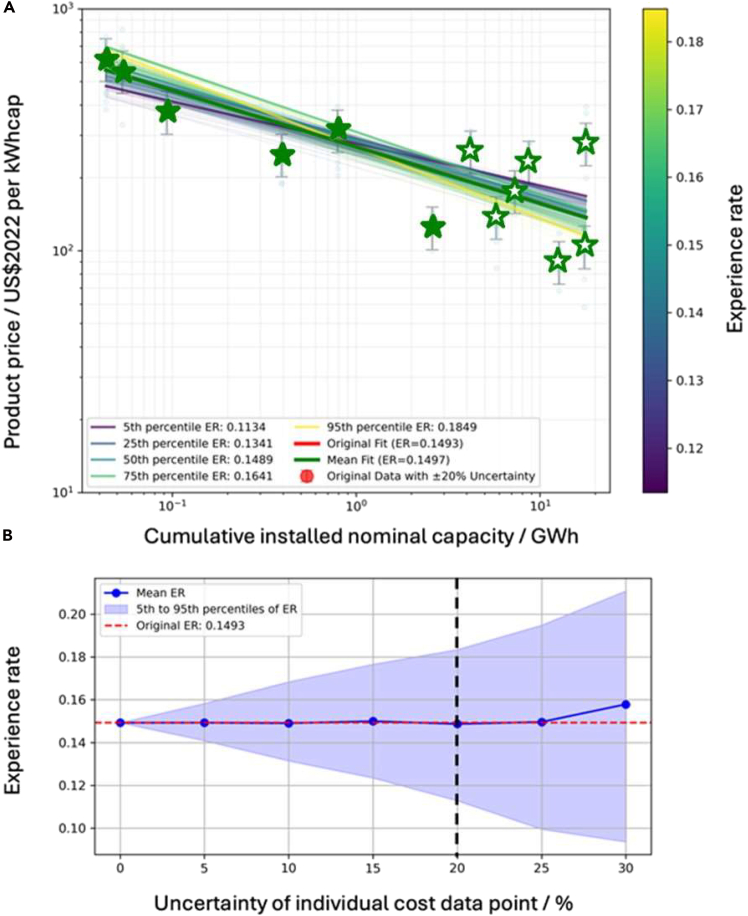
Uncertainty analysis of the cost data (A) Wright’s Law curve fits under ±20% data uncertainty. (B) Sensitivity analysis of the experience rate as the assumed uncertainty in each cost data point increases from 0% to 30%. The Mean ER (blue line) remains nearly constant, demonstrating the robustness of the technological trend, while the shaded area represents the expanding 5th–95th percentile confidence interval as data reliability decreases.

***Note:*** See troubleshooting 2.

4. Perturb costs and refit repeatedly.
  a. Randomly perturb each *P*(*X*) within the predefined error band using a uniform distribution to create one synthetic cost sample.
    ***Note:*** Assume that cost errors are independent across different data points to avoid unobserved correlations.
  b. Refit Wright’s Law as in Major Step 1 to obtain one ER draw.
  c. Repeat a–b for N trials to build an ER sample distribution.
    **CRITICAL:** Keep the same perturbation rule, error band, and random-seed setting across trials to avoid introducing extra bias.
5. Export ER and future-cost uncertainty bands.
  a. Report ER mean/median and confidence.
  b. Propagate each trial’s fit forward to obtain future cost bands (Figure 3).

### Embed cost into DCF to evaluate financial viability by duration

**Timing: 1 month**

This final section focuses on evaluating the financial feasibility of storage projects using a life-cycle DCF model. It integrates the projected cost declines with revenue structures to align the Project Value-to-Risk (PVR) with the Total Cost of Ownership (TCO), facilitating an assessment of the technology’s long-term economic viability.

This major step feeds the cost into a lifecycle DCF model and evaluates feasibility by aligning PVR with TCO across discharge-duration scenarios.

6. Load DCF parameters and scenarios.
  a. Import lifecycle inputs (project life, discount rate, RTE, fixed/variable O&M) and revenue structure (arbitrage, capacity payments, etc.).
  b. Import discharge-duration scenarios (e.g., 3–1000 h).

***Note:*** See troubleshooting 3.

7. Compute PVR and TCO for each scenario.
  a. Choose a specific duration scenario.
  b. Calculate PVR and TCO over the lifecycle.
  c. Repeat steps a and b for all remaining duration scenarios.

***Note:*** The following formulation describes the energy-normalized DCF equation and its constituent variables^3^:

$$\begin{array}{l} \sum_{t=1}^{T}{\left(1+r\right)}^{-t}\left[\Delta_{E,t}{n_{c}}_{,t}+{R_{P}}_{,t}d^{-1}\right]=C_{P}d^{-1}+{C_{E}}_{,th}\eta_{D}^{-1}+\sum_{t=1}^{T}{\left(1+r\right)}^{-t}\left[{n_{c}}_{,t}{P_{C}}_{,t}\left({{\eta_{R}}_{TE}}^{-1}-1\right)+{n_{c}}_{,t}VOM_{t}+d^{-1}FOM_{t}\right]+{C_{R}}_{e}{\left(1+r\right)}^{-L/n_{c}} \end{array}$$

where, $\sum_{t=1}^{T}{\left(1+r\right)}^{-t}$ is summation over the project term *T*, discounting each term by the discount rate *r*, Δ_*E*,*t*_ is frequency and average price differential of charging and discharging modes (indicative of arbitrage applications), ${n_{c}}_{,t}$ is number of cycles in year *t*, ${R_{P}}_{,t}$ is revenue from capacity payments in year *t*, *d* is duration at rated power, *C*_*p*_ is installed power cost, *C*_*E,th*_ is theoretical installed energy cost (without factoring in efficiency losses and depth of discharge limitations), *η*_*D*_ is discharge efficiency, *P*_*c*_ is charging electricity price per cycle, ${\eta_{R}}_{TE}$ is round-trip efficiency, VOM is variable operating and maintenance cost, FOM is fixed operating and maintenance cost, *C*_*Re*_ is replacement cost of the energy storage medium, and L is cycle life of the storage system.

**Figure 4:**
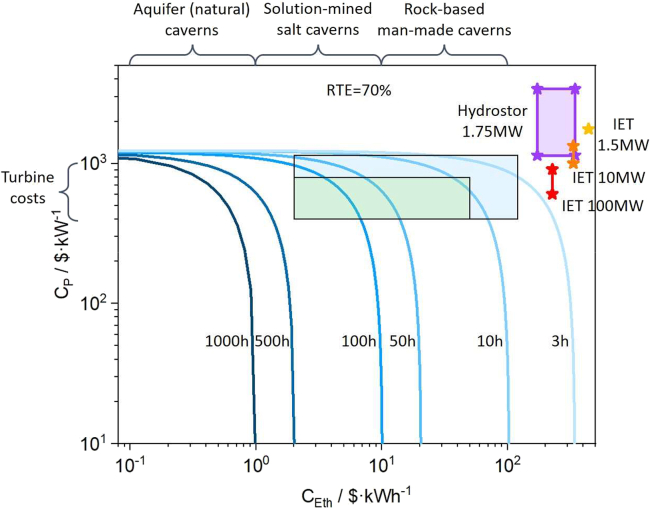
Economics of A-CAES for long-duration energy storage: Power and energy cost trends by discharge duration

***Note:*** Repeat Steps 6–8 under alternative market price or policy assumptions if needed.

8. Identify feasibility windows and cost-reduction potential.
  a. Determine the durations/years where *PVR* ≥ *TCO*.
  b. Report feasibility boundaries and sensitivity results (Figure 4).

## Expected outcomes

This protocol is expected to produce a reproducible set of learning-curve and financial-viability outputs for CAES. Using the harmonized project database, the Wright’s Law analysis will generate the following deliverables: 1) Fitted Parameters: Precise experience rates (ER) and regression coefficients. 2) Cost Trajectories: Projected unit cost declines modeled against cumulative capacity. 3) Statistical Distributions: Uncertainty intervals and fit diagnostics (e.g., R^2^, slope). 4) Feasibility Boundaries: The operational limits and economic thresholds for system deployment. When observational/measurement error is introduced, the Monte Carlo stage should generate a probability distribution for ER (such as mean/median with a 5th–95th percentile range) and corresponding uncertainty bands for future cost projections, allowing researchers to quantify how robust the inferred learning trend is to data noise. Embedding the cost into a lifecycle DCF model should then produce duration- and scenario-specific PVR–TCO comparisons, including feasibility thresholds (the discharge durations/years where *PVR* ≥ *TCO*), how feasibility evolves over time as costs fall, and sensitivity rankings for key economic inputs (e.g., discount rate, price spread, RTE, O&M, and capacity payments). The final research deliverables are exemplified in Figures 1, 2, 3, and 4 to illustrate the potential format of the results.

## Limitations

The limitations of this protocol are categorized into inherent methodological constraints and context-dependent factors that users may partially mitigate. First, inherent limitations of the protocol are intrinsic to the chosen mathematical models and the nature of the CAES industry; they represent boundaries that users should acknowledge when interpreting results. Wright’s Law assumes cost decline is primarily driven by cumulative deployment. However, CAES costs are also shaped by exogenous drivers (e.g., site-specific geology, permitting complexity, and supply-chain volatility). When these factors dominate, experience-curve extrapolation may misestimate the true pace of cost reduction. Besides, the DCF results are inherently sensitive to market-driven inputs such as discount rates and price spreads. Similarly, the Monte Carlo stage relies on assumed error distributions; if real-world uncertainties contain structural biases (e.g., systematically misreported pilot costs), the resulting intervals may be miscalibrated. Second, context-dependent limitations depend on the specific data environment and user configuration. Users can partially mitigate these effects through rigorous data handling and parameterization. CAES projects are few, and reported costs often use inconsistent boundaries (e.g., CapEx vs. EPC). While the protocol harmonizes currencies and applies scale-based screening, users can further mitigate bias by ensuring high-quality, primary data sources and strictly aligning cost boundaries before input. Additionally, feasibility boundaries are highly dependent on regional market-sensitive inputs (e.g., capacity payments, RTE, O&M). Users must re-parameterize the model for their specific target market. The protocol is designed to be flexible; accurate local parameterization will refine the relevance of the feasibility boundaries.

## Troubleshooting

### Problem 1

The experience-curve regression yields an implausible slope or very low fit quality.

This is usually caused by mixed cost boundaries (CapEx vs. EPC vs. total installed cost), incomplete currency/year harmonization, or a high-leverage outlier (often early pilot projects).

### Potential solution

• Recheck that all costs are converted to the same base year and unit (e.g., 2022 USD/kWh) and cumulative capacity is in GWh.
• Move pilot-scale or boundary-uncertain projects into a separate subset and refit both the full sample and a ≥10 MW subset.
• Run leave-one-out checks to identify single-point leverage.

### Problem 2

Monte Carlo simulation outputs show unrealistically narrow or wide uncertainty bands for the experience rate (ER) and future cost projections.

This is typically caused by improper assumptions regarding the input probability distributions, a lack of correlation between perturbed cost points, or an insufficient number of simulation trials failing to achieve statistical convergence.

### Potential solution

• Refit the input error distributions (e.g., testing normal vs. uniform) to ensure they adequately capture tail risks and verify that the baseline uncertainty percentage (e.g., ±20%) aligns with historical data volatility.
• Increase the number of Monte Carlo trials to at least 10,000 and plot the cumulative mean of the ER to ensure the simulation has converged to a stable value.
• Vary the assumed uncertainty range (e.g., from 0% to 30%) and document how the width of the cost trajectories shifts to identify if the model is overly sensitive to specific data clusters.

### Problem 3

DCF parameters (discount rate, price spread, capacity payments, RTE, O&M, etc.) are inaccurate or not market-consistent, which distorts PVR/TCO and feasibility boundaries.

DCF outcomes are highly market- and policy-sensitive, and these inputs vary widely across regions and time; using non-target-market assumptions can systematically bias conclusions.

### Potential solution

• Collect the latest 12-month energy market data (especially price spreads, capacity payments, discount rates, and efficiency) and re-run the DCF model using these target-market constants.
• Vary one parameter at a time (OAT) within a predefined credible range (e.g., ±20%) and document the resulting shift in PVR/TCO to identify primary cost drivers.

## Resource availability

### Lead contact

Further information and requests for resources should be directed to and will be fulfilled by the lead contact, Wei He (wei.4.he@kcl.ac.uk).

### Technical contact

Further technical information should be directed to and will be fulfilled by the technical contacts, Wei He (wei.4.he@kcl.ac.uk) and Danlei Yang (danlei.yang2023y@gmail.com).

### Materials availability

This study did not generate unique materials.

### Data and code availability

• All data supporting the findings of this study are available upon reasonable request.
• The codes used in the discounted cash flow analysis are listed in the key resources table, which are available at Zenodo: https://doi.org/10.5281/zenodo.17194806.
• Additional related information is listed in the key resources table and is available from the lead contact upon reasonable request.

## Acknowledgments

We would like to acknowledge the support of UKRI-EPSRC (grant no. EP/W027372/1). W.H. would like to acknowledge the support of the Royal Academy of Engineering (RAEng) Engineering for Development Research Fellowship (grant no. RF∖201819∖18∖89).

## Author contributions

D.Y. contributed to conceptualization, methodology, software, validation, formal analysis, investigation, data curation, visualization, writing – original draft, and writing – review and editing. Y.W. contributed to investigation and writing – review and editing. J.W. contributed to project administration and funding acquisition. Z.R. contributed to investigation and writing – review and editing. W.H. contributed to conceptualization, methodology, investigation, resources, visualization, writing – original draft, writing – review and editing, supervision, project administration, and funding acquisition.

## Declaration of interests

The authors declare no competing interests.
